# Detection of congestive heart failure from RR intervals during long-term electrocardiographic recordings

**DOI:** 10.1016/j.hroo.2025.01.014

**Published:** 2025-01-31

**Authors:** Teemu Pukkila, Matti Molkkari, Jussi Hernesniemi, Matias Kanniainen, Esa Räsänen

**Affiliations:** 1 Computational Physics Laboratory, Tampere University, Tampere, Finland; 2 Finnish Cardiovascular Research Center Tampere, Tampere University, Tampere, Finland; 3 Heart Hospital, Tampere University Hospital, Tampere, Finland; 4 Faculty of Medicine and Health Technology, Tampere University, Tampere, Finland

**Keywords:** Congestive heart failure, Electrocardiography, Holter monitoring, Heart rate variability, Detrended fluctuation analysis

## Abstract

**Background:**

Timely detection is crucial for managing cardiovascular diseases. Recently developed computational tools to analyze RR interval (RRI) sequences offer cost-effective means for early cardiac screening and monitoring with consumer-grade heart rate devices.

**Objective:**

The purpose of this study was to demonstrate detection of congestive heart failure (CHF) from RRIs by discriminating CHF from both healthy controls and patients with present atrial fibrillation (AF). We also examined the detection’s consistency regarding CHF severity and AF episode frequency.

**Methods:**

We analyzed RRIs extracted from several datasets of long-term electrocardiographic (ECG) recordings. We use detrended fluctuation analysis (DFA) to evaluate the correlations of RRI, that is, how changes in the RRIs affect changes at another time. Furthermore, we utilized dynamical detrended fluctuation analysis (DDFA), which provides further insights into how the correlations change over time and different time scales. The resulting (D)DFA scaling exponents are used as features in classification, distinguishing CHF, AF, and healthy controls using the XGboost ensemble learning technique.

**Results:**

Our (D)DFA computations revealed distinct RRI characteristics for CHF and AF patients during long-term ECG recordings, aiding disease detection. The DDFA-based classification pipeline detects CHF/AF from healthy controls with 90% sensitivity and 92% specificity. The 3-class classification algorithm correctly detects 78% of AF cases, 78% of CHF cases, and 91% of healthy cases. The DDFA results show consistency regarding CHF severity and AF episode frequency.

**Conclusion:**

We achieved high confidence in detecting CHF, with DDFA showing excellent classification accuracy, especially in multiclass tasks. This approach highlights the potential of noninvasive, cost-efficient RRI analysis for early detection of CHF and AF.

**Key Findings**

▪ The study achieved 90% sensitivity and 92% specificity in distinguishing congestive heart failure (CHF) and atrial fibrillation (AF) from healthy controls using dynamical detrended fluctuation analysis (DDFA) utilizing only the RR intervals during a 24-hour recording.
▪ DDFA revealed unique RR interval characteristics for CHF and AF patients, assisting in accurate classification of these conditions. This method provides detailed information based on heart rate and scale, capturing complex patterns in the RR interval dynamics, which conventional heart rate variability measures might miss.
▪ The approach highlights the potential of using noninvasive, cost-efficient RR interval analysis for early detection of CHF and AF, which could be implemented in consumer-grade heart rate devices for remote monitoring.

## Introduction

Heart failure is globally the leading cause of morbidity and mortality,^1^ and congestive heart failure (CHF) has an estimated prevalence of 26 million people globally.^2^ Echocardiography is essential for assessing ventricular function and thus usually is performed in patients with suspected CHF. This contributes to high health care costs, and the problem will only worsen with an aging population.^3^ Therefore, cost-effective and reliable screening of CHF is important to monitor the high-risk population. In this study, we investigated the detection of CHF with advanced heart rate (HR) variability (HRV) measures against healthy controls and subjects with present atrial fibrillation (AF), which is the most common type of cardiac arrhythmia with an estimated prevalence of more than 59 million individuals worldwide.^4^

Measuring interbeat intervals and HRV has become more common in everyday life, and consumer-grade devices are capable of accurately collecting long-term interbeat and/or RR interval (RRI) time series.^5^^,^^6^ Smartwatches assess sleep, fitness, and recovery with HRV and even detect cardiac diseases such as AF.^7^ There is an increasing market and need to detect other cardiovascular diseases, and in principle HRV measurements may provide noninvasive and cost-effective tools for this purpose. Unfortunately, however, most of the present HRV measures do not provide sufficiently accurate information to meet the diagnostic needs. Some studies show good classification accuracy by combining multiple HRV measures to compare CHF patients and healthy controls.^8^^,^^9^ However, the conventional HRV measures do not provide insight into the behavior of the heart across different cardiac conditions. Furthermore, previous studies have a few limitations. First , the robustness of the classification algorithms have not been rigorously tested, and proper assumptions about the generalization of the models outside the very limited and specific datasets are not well justified. Second, these “black box” models utilizing multiple measures as features do not properly provide insight into the behavior of the heart for different pathologic cases.

A promising nonlinear HRV method for detecting cardiovascular diseases is detrended fluctuation analysis (DFA),^10^^,^^11^ which already has been used to show differences between healthy controls and CHF patients regardless of preserved ejection fraction.^12^ In recent years, use of DFA has been extended to cover accurate scale dependency^13^ and dynamical analysis.^14^ These latest advancements have provided promising results in predicting long QT syndrome (LQTS).^15^ Moreover, a very recent study of 2794 subjects from the FINCAVAS (Finnish Cardiovascular Study) database with mean follow-up duration of 8 years found that scale-dependent DFA can assess the risk for sudden cardiac death with excellent confidence (hazard ratio ≈2. 5) with beat-to-beat (RR) intervals extracted from a 1-minute rest measurement before the cardiac stress test.^16^

In this study, we utilized an advanced time-series analysis method called dynamical detrended fluctuation analysis (DDFA).^14^ We use DDFA to examine the characteristics of the RRI time series, in particular, the nature of the correlations between the RRIs at different time scales and as functions of time and HR, respectively. The resulting DDFA scaling exponents describing the correlations [ie, α(t,s) and α(HR,s)] reveal unique behaviors for different cardiac conditions. We focused on the specific changes observed in the DDFA scaling exponents for CHF patients, which also were compared to patients with AF.

## Methods

### Data

We used multiple databases from both PhysioNet (The Research Resource for Complex Physiologic Signals)^17^ and the Telemetric and Holter ECG Warehouse (THEW).^18^^,^^19^ All the databases with their sample sizes are listed in Table 1. Note that the number of subjects in the *definite* database differs from the original source because of missing electrocardiographic (ECG) data (N = 16), exclusion of subjects with both AF and CHF (N = 20), and 1 subject with insufficient ECG data quality. The duration of the recordings is approximately 24 hours except for *mitafdb,* which has 10-hour recordings. We combined the individual datasets to a pooled sample consisting of 530 subjects. Of these subjects, 147 individuals were diagnosed with CHF, and 109 had AF episodes during ECG recording. As healthy controls, 274 participants who had no cardiovascular diseases or arrhythmias were used. All the databases used in this study contain only de-identified data, and the study was conducted in accordance with the principles outlined in the Helsinki Declaration.

**Table 1:** Databases, their abbreviations, and the number of samples

Database	Abbreviation	N (M/F)	Age, y	HR, bpm
MIT-BIH Normal Sinus Rhythm^20^	nsrdb	18 (5/13)	34±8	78±7
Normal Sinus Rhythm RR Interval^21^	nsr2db	54 (30/24)	61±12	80±8
MIT-BIH Atrial Fibrillation^22^^,^^23^	mitafdb	25 (–/–)	—	84±16
Long Term AF^24^^,^^25^	ltafdb	84 (–/–)	—	83±17
BIDMC Congestive Heart Failure^26^^,^^27^∗	chfdb	15 (11/4)	56±11	91±15
Congestive Heart Failure RR Interval^28^†	chf2db	29 (8/4)‡	55±12	90±11
Healthy E-HOL-03-0202-003^29^§	thew	202 (102/100)	38±16	80±10
DEFINITE E-HOL-03-0401-017^30^‖	definite	103 (75/28)	56±13	83±17

Age and heart rate (HR) expressed as mean ± SD.

F = female; M = male.

∗ In the chfdb database, all subjects are classified as New York Heart Association (NYHA) class III or IV.

† In the chf2db database, the distribution of subjects by NYHA class is I (4 subjects), II (8 subjects), and III (17 subjects).

‡ The chf2db database includes 21 subjects with unspecified sex.

§ For the thew database, the average body mass index is 24 ± 4 kg/m^2^, with systolic blood pressure of 117 ± 13 mm Hg and diastolic blood pressure of 75 ± 8 mm Hg.

‖ For the Definite database, the average body mass index is 30±7 kg/m^2^, with a left ventricular ejection fraction of 21 ± 6%. The distribution of subjects by NYHA class is: I (20 subjects), II (64 subjects), and III (19 subjects).

In the analysis, we focused on 3 different groups; healthy controls (*nsrdb*, *nsr2db*, *thew*); AF (*mitafdb*, *ltafdb*); and CHF (*chfdb*, *chf2db*, *definite*).

The PhysionNet databases contain RRIs as annotations, which are automatically detected by PhysioToolkit.^17^ For the THEW database, we used an in-house R peak detection algorithm, which was used also in other studies.^15^^,^^31^ In preprocessing, we removed the RRIs that could be considered statistical outliers in the surrounding context of the RRI time series. Of note, we did not attempt to filter the data on physiological criteria, such as non-sinus beats, which contain information about different cardiac conditions.

Filtering is implemented by applying a rolling median filter to the data, in which RRIs are discarded if they fall outside of 0.75η≤RRI≤1.5η, where η is the local median with a kernel of 31 beats. Moreover, RRIs also are discarded based on the difference between subsequent intervals with a threshold value of 200 ms. Similar preprocessing has also been used in previous studies.^14^^,^^15^

### DDFA

DFA originally was developed to study long-range power-law correlations in DNA sequences,^10^ but it has been applied to various time series in different fields. In RRI analysis, it was shown early on that DFA displays different results for healthy and pathologic cases.^11^ The key output of DFA—the scaling exponent α—describes the *collective correlations* of the studied RRI time series in contrast to the *pointwise correlations* of the autocorrelation function. Conventionally, α has been split into short-scale $\alpha_{1}$ (scales 4–16) and long-scale $\alpha_{2}$ (scales 16–64). These values are computed over the whole RRI time series.

DFA has been extended to a robust scale-dependent examination^13^ and later DDFA that yields the dynamical landscapes of time- and scale-dependent scaling exponents α(t,s).^14^ These extensions and further innovations have been crucial in extending the accuracy and practical applicability of DFA to examine the physiological condition of subjects.^32^ Recent applications utilizing the new features and extensions of DFA include the prediction of sudden cardiac death,^16^ estimation of physiological exercise thresholds,^33^ detection of LQTS,^15^^,^^33^ and effects of hypoxia on cardiac cells.^34^

The conventional DFA algorithm uses linear detrending (DFA-1). Here we applied second-order polynomials in the detrending (ie, DFA-2). This method has been shown to overperform DFA-1 in detecting LQTS patients^15^ and in predicting sudden cardiac death.^16^ We utilized 86 logarithmically distributed scales between 5 and 1000 for the DDFA α(t,s) calculations. These scaling exponents can be further aggregated as a function of other variables. Here we used the HR that has the second variable besides the scale [ie, α(HR,s)]. This is performed in 190 individual HR bins in the range 30–220 bpm by grouping α(t,s) according to the mean HRs. To supplement the analysis, we also examined the *density* of the scaling exponents, ρ(α,s). It is computed independently for each scale s by using 50 uniformly spaced bins for α(t,s) in the range from 0–2.

## Classification

For the classification task, we used an XGBoost ensemble model,^35^ complemented by dimensionality reduction using principal component analysis (PCA). The classification is performed separately for both DFA and DDFA features. For DFA, we utilized both DFA-1 and DFA-2 scaling exponents as features. For DDFA, we leveraged α(HR,s) and ρ(α,s) surfaces, which are resized into X-by-Y images using bilinear interpolation for inclusion as features. Notably, the absolute HR range varies from person to person due to factors such as physical activity, medications, and health. To account for this variability during classification, we imputed the empty HR and scale bins as 0, corresponding to the scaling exponent α value of a constant signal. In addition, a few basic HR features from the filtered time series were included into the analysis: mean HR, its standard deviation, and the 1st, 25th, 75th and 99th percentiles of the HR during the measurement. The α(HR,s) surfaces are split into rest and exercise, containing HRs below and above 100 bpm, respectively. This division allows for different resizing of the rest and exercise segments, resulting in an improved feature space.

**Figure 1:**
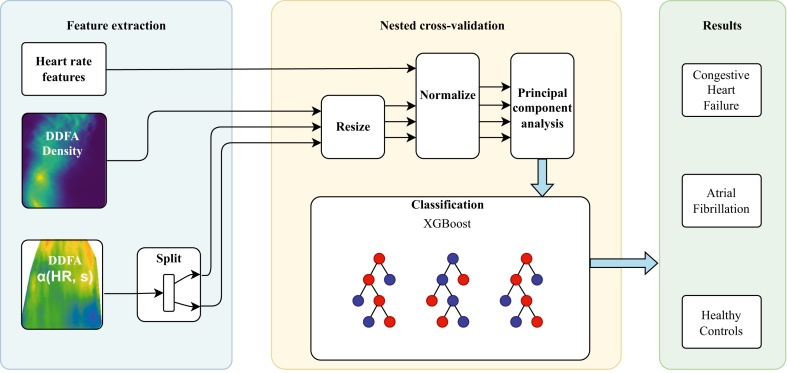
Illustration of the classification pipeline. [For more details about dynamical detrended fluctuation analysis (DDFA) density and DDFA α(HR, *s*), see Figure 5.]

The classification pipeline for the DDFA classifier is shown in Figure 1. For DFA classification, a similar pipeline is used, but because of the simplicity of the features, only PCA and XGBoost are necessary. In the case of DDFA, the features exhibit widely varying value ranges. Therefore, each feature is normalized using MinMaxScaler, ensuring that the smallest value maps to 0 and the largest to 1.

The model performance was evaluated using nested cross-validation, which involved 10 folds of train/test splits with a 50% test size. For each fold, we performed conventional cross-validation with 10 additional folds. Optimal parameters for resizing, PCA, and XGBoost were chosen independently in each conventional cross-validation step using grid search. The parameter space details are given in Supplemental Table 1.

## Results

**Figure 2:**
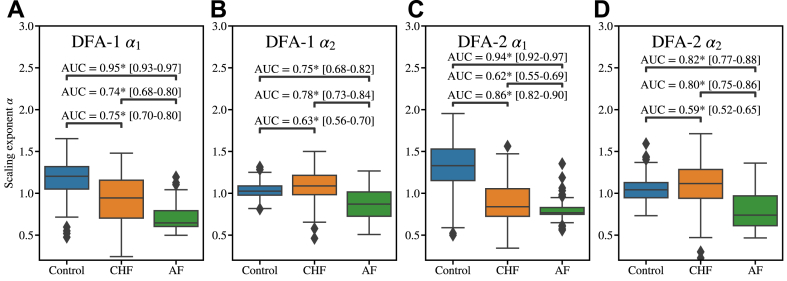
Scaling exponents of detrended fluctuation analysis (DFA) for each of the studied subgroups and the receiving operating characteristics area under curve (AUC) values between the different subgroups. **a**: First-order DFA (DFA-1) short-scale scaling exponent α_1_. **b:** DFA-1 long-scale scaling exponent α_2_. **c:** Second-order DFA (DFA-2) short-scale scaling exponent α_1_. **d:** DFA-2 long-scale scaling exponent α_2_. *Square brackets* indicate 95% confidence intervals for the AUC values estimated by DeLong’s algorithm.^36^ Statistically different AUC values from 0.5 are indicated by *asterisks.* AF = atrial fibrillation; CHF = congestive heart failure.

Figure 2 shows the DFA scaling exponents for different study groups (Healthy, CHF, and AF) with area under the curve values indicating the distinguishability between these groups. The results are shown for first-order and second-order detrending and for both short- and long–range exponents $\alpha_{1}$ and $\alpha_{2}$, respectively. As the most distinctive observation, $\alpha_{1}$ has significantly lower values in pathologic cases (CHF and AF) compared to healthy controls. This is in line with several previous studies.^11^, ^12^, ^13^^,^^15^ In contrast, the $\alpha_{2}$ values do not exhibit similar distinction; however, all the area under the curve values still are statistically significant. Second-order detrending in DFA-2 further enhances the differences found with DFA-1. In particular, the difference between CHF and healthy increases. This is consistent with previous findings of some of the present authors in assessing the HRV characteristics of patients with LQTS^15^ and risk for sudden cardiac death.^16^

The effect of age and sex on DFA was assessed using multivariable logistic regression (MLR) to compare CHF and control groups, with a primary focus on the short-scale scaling exponent $\alpha_{1}$, as it demonstrates significantly better discriminatory power between CHF and control groups than $\alpha_{2}$. Table 2 shows the MLR results from DFA-2 $\alpha_{1}\text{,}$ and Supplemental Tables 2–4 show the equivalent results for other detrending orders and long-range scaling exponents. Both DFA-1 and DFA-2 $\alpha_{1}$ were statistically significant (*P* <.001) in the MLR analysis with large negative logistic regression coefficients, indicating a strong inverse association with the outcome. In contrast, age did not reach statistical significance (*P* >.05), suggesting a minimal effect, whereas sex was statistically significant but to a lesser extent than DFA, indicating that it may have a modest confounding influence. In the case of $\alpha_{2}$ both age and sex showed statistical significancy in the model, but the model has low predictability with pseudo R^2^ values of 0.13 and 0.32 for DFA-1 $\alpha_{2}$ and DFA-2 $\alpha_{2}$, respectively.

**Table 2:** Results of the logistic regression model examining the relationship between the predictor variables and the dependent binary outcome

Variable	Coeff. (95% CI)	SE	z	Pvalue
Constant	4.6737 (1.484 to 7.864)	1.627	2.872	.004
DFA-2 $\alpha_{1}$	–7.1774 (–9.721 to –4.634)	1.298	–5.530	.001
Age	0.0131 (–0.021 to 0.048)	0.018	0.744	.457
Sex	–1.6811 (–2.903 to –0.459)	0.624	–2.696	.007

Estimated coefficients (Coeff.) along with their 95% confidence intervals (CIs), the standard error of the estimates (SE), the z-statistics (z) for testing whether the coefficient is significantly different from 0, and the associated *P* values. Significant predictors are highlighted by *P* <.05. Positive coefficients indicate an increase in the log odds of the outcome with an increase in the predictor, whereas negative coefficients indicate a decrease.

**Figure 3:**
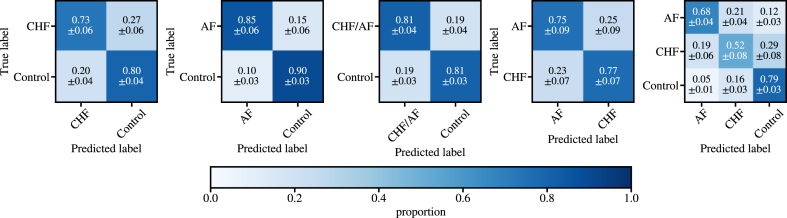
Confusion matrices for the XGBoost classifier with congestive heart failure (CHF), atrial fibrillation (AF), and healthy controls. The classification features include the short- and long-scale scaling exponents of both the first- and second-order detrended fluctuation analysis.

The confusion matrices resulting from the classification are shown in Figure 3. We point out that in addition to binary classification between 2 groups (CHF and AF), we extend the classification task to a multiclass problem, aiming to identify not only whether a patient had CHF or AF but also the specific disease type (CHF vs AF vs healthy). CHF binary classifier results in sensitivity of 73% and specificity of 80%, whereas results for the AF classifier are 85% and 90%, respectively. Our findings indicate that the distinction between control and CHF/AF cases is relatively accurate for such a simple model, with sensitivity of 81% and specificity of 81%.

However, both the binary classifier between AF and CHF and the multiclass classifier struggle to differentiate CHF from AF subjects, often misclassifying CHF as AF and vice versa. In the binary classifier, where AF is presented as the positive and CHF as negative class, the model reaches sensitivity of 75% and specificity of 77%. In the multiclass task, we find a balanced accuracy of 66%. These results reinforce our conclusion that conventional DFA (including DFA-1, DFA-2, $\alpha_{1}$, and $\alpha_{2}$) can detect differences between healthy and pathologic cases but lacks sufficient specificity to accurately differentiate pathologic conditions due to its inherent simplicity.

**Figure 4:**
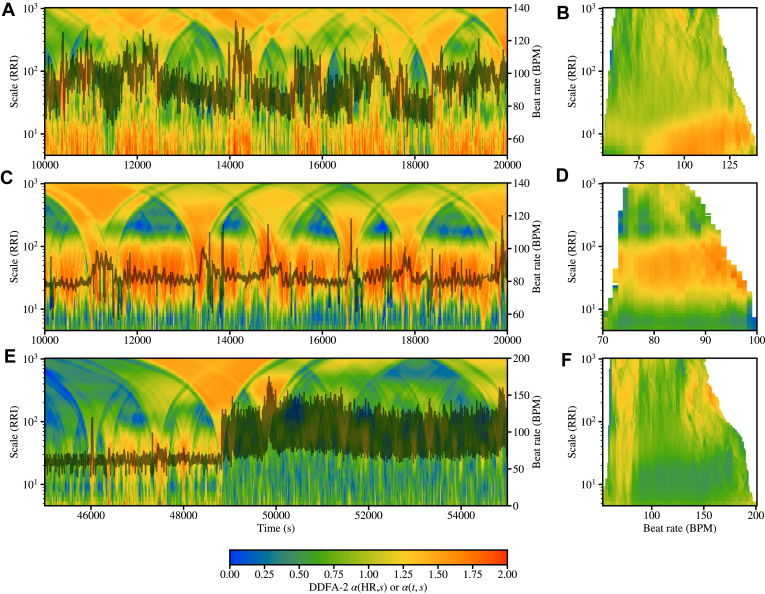
Examples of scaling exponent landscapes (in color) α(*t*, *s*) **(a, c, e),** and α(HR, *s*) **(b, d, f)** computed with dynamical detrended fluctuation analysis for RR intervals collected from a healthy subject **(a, b),** congestive heart failure patient **(c, d),** and atrial fibrillation patient **(e, f).**

Next we focus on DDFA that supplements the analysis by incorporating the time- and scale-dependent characteristics of the scaling exponent. Figure 4 shows examples of α(t,s) and α(HR,s) landscapes for a healthy control (a, b), CHF subject (c, d), and AF subject (e, f). The differences between the healthy and CHF/AF subjects are evident in the HR dynamics. In CHF, the local HR variations are distinctively small compared to that of the healthy, whereas in the AF subject the onset of the episode at about 49,000 seconds is evident, characterized by very strong variations.

DDFA scaling exponents displayed by the color landscapes reveal further differences between the subjects (Figure 4). Regarding CHF, the most striking feature is the shift of the band of high α values to higher scales, with lower values both above and below this band. On the other hand, the AF subject during the episode is characterized by low α values in an extensive range of scales. In addition, the phase before onset of the AF episode is characterized by considerably small α values in small scales compared to the healthy example.

**Figure 5:**
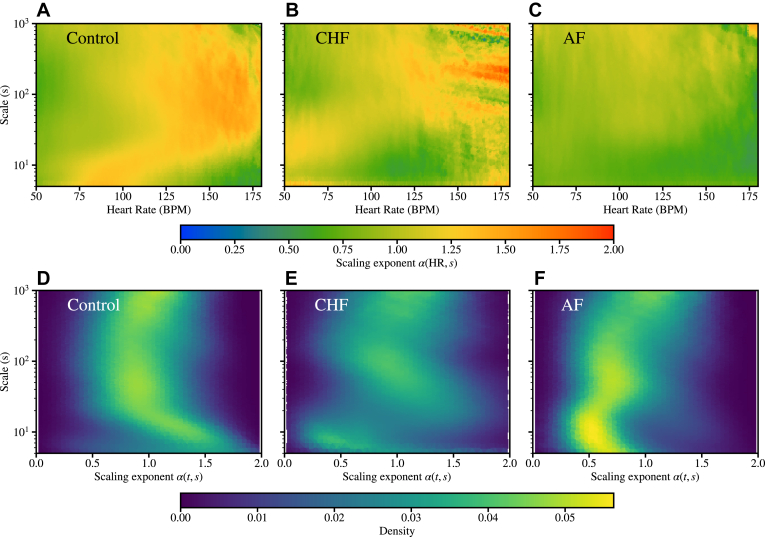
Aggregated plots of the scaling exponents α(HR, *s*) (in color) as functions of the scale *s* and heart rate (HR) resulting from dynamical detrended fluctuation analysis. **a:** All the healthy controls. **b:** All the congestive heart failure (CHF) subjects. **c:** All the atrial fibrillation (AF) subjects. The HR range is limited to 50–180 for visual purposes. **d–f:** Corresponding aggregated densities of the scaling exponent.

Figure 5 shows aggregated α(HR,s) across *all* the subjects in the control (a), CHF (b), and AF (c) groups, respectively. The aggregates show the mean values over all the subjects, where each subject is represented by the mean values of each HR bin. Thus, each subject is weighted equally in the aggregate plot.

We first point out that CHF patients do not achieve high HRs during 24-hour Holter monitoring. The α(HR,s) values above HR=150 in Figure 5b are due to a few outliers in the CHF data. As a distinct feature, healthy subjects exhibit significantly higher α(HR,s) values than the CHF subjects at low HRs and small scales. In particular, at around HR=75…100 and scales s=5…15, the healthy and CHF subjects show α=1.2…1.4 and 0.7…1, respectively. In contrast, AF patients display relatively uniform α values around 0.75–1.0 across an extensive range of HR and s.

These findings in Figures 5a–5c suggest that the differences in the scaling exponents at low scales and low HRs effectively distinguish healthy controls from the pathologic cases. However, CHF and AF exhibit distinct behaviors in the high HR range, with CHF displaying patterns similar to healthy controls, except for the absence of very high HRs. The classification power of the results is analyzed in the following.

In Figures 5d––5f we show the aggregated *densities* of the scaling exponents, that is, α distributions, for the control, CHF and, AF groups, respectively. This provides an alternative view on the differences in the scaling exponents between the groups. At small scales (s≲20), the healthy subjects show relatively high densities at high α alpha values, which, as expected, is in contrast with the low densities displayed by the CHF and AF groups. On the other hand, the shapes of the density distributions are distinct. The healthy subjects exhibit a characteristic “C”-shaped cloud, whereas CHF and AF patients display more " ϵ"-shaped distributions, where the density as function of the scale fluctuates more than that of the healthy controls. The most notable differences between the CHF and AF groups are the higher densities of the AF scaling exponents at intermediate/large scales (20≲s≲100) and low α values, whereas the CHF densities are more evenly spread into higher α values. In AF, the density is also strongly concentrated along the " ϵ"-shaped path on the (α, s) plane. This effect is likely due to the fragmentation of the AF data in terms of AF episodes and normal rhythm, an effect analyzed in more detail in the following.

**Figure 6:**
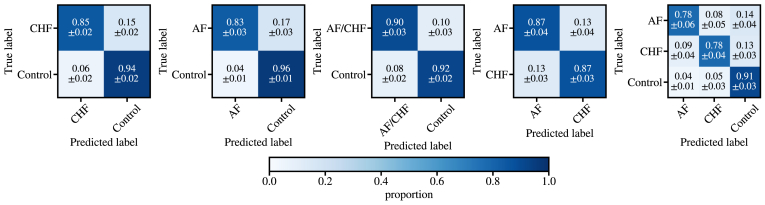
Confusion matrices for the XGBoost classifier with congestive heart failure (CHF), atrial fibrillation (AF), and healthy controls. The classification features are extracted from dynamic detrended fluctuation analysis with principal component analysis dimensionality reduction.

Figure 6 shows the confusion matrices for the DDFA features using an XGBoost classifier with PCA applied resized DDFA images. Compared to the analysis with conventional DFA, the additional information given by DDFA significantly increases both sensitivity and specificity in the binary classification cases. An exception is the comparison between AF and the control group, where the DDFA model has only 2% unit decrease in sensitivity but 6% unit increase in specificity compared to the DFA model. The most significant improvements are observed in the CHF vs Control classification, achieving 85% sensitivity and 94% specificity, representing increases of 12 percentage points in sensitivity and 14 percentage points in specificity compared to conventional DFA. In the multiclass case, the balanced accuracy is increased from 66% to 82% with DDFA, and correctly classified CHF patients increase from 52% to 78%. The false-negative rates, where CHF or AF cases are misclassified as healthy, are 14% for CHF and 13% for AF. In comparison, the false-positive rates are notably lower: 5% for CHF and 4% for AF.

**Figure 7:**
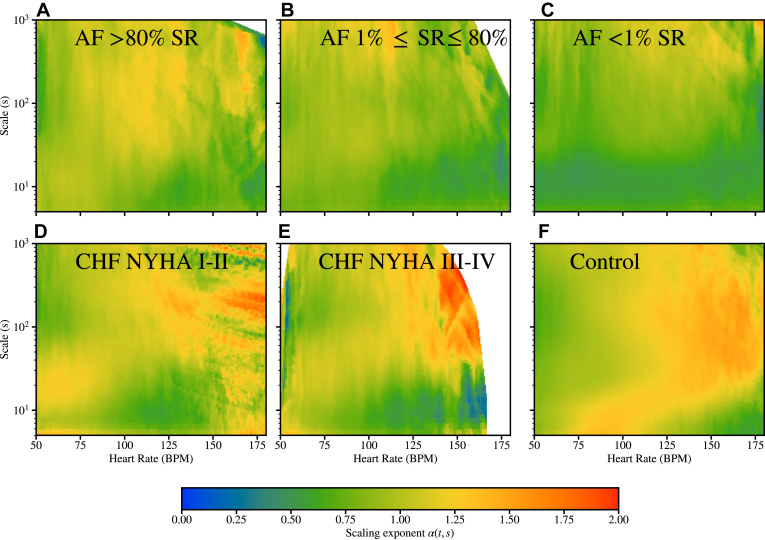
Aggregated plots of the scaling exponents α(HR, *s*) (in color) as functions of the scale *s* and heart rate (HR) resulting from dynamical detrended fluctuation analysis. The results are grouped based on the severity of the condition. **a:** Atrial fibrillation (AF) with >80% of the measurement duration in sinus rhythm (SR) (N = 26). **b:** AF with 1%–80% in SR (N = 24). **c:** AF with <1% in SR (N = 34). **d:** Congestive heart failure (CHF) with New York Heart Association (NYHA) class I or II (N = 96). **e**: CHF with NYHA class III or IV (N = 51). **f:** Healthy controls (N = 274).

Finally, we extend our analysis into distinctive subgroups of CHF and AF by considering the severity of CHF in terms of the New York Heart Association (NYHA) class and the frequency of AF episodes during the measurements. The CHF dataset is split into mild (NYHA class I and II) and severe (NYHA class III and IV) subgroups. In addition, the *ltafdb* AF data are split into 3 groups based on the relative proportion of the measurement time spent in sinus rhythm (SR): 80% in SR; 1%−80% in SR, and 1% in SR. Figures 7a–7c shows that even though the increasing proportion of non-SR (AF episodes) leads to reduced α values, the patients with >80% of time in SR still exhibit noticeable differences from the healthy controls (Figure 7f). This observation (to be analyzed further in a separate study) gives promising prospects in using DDFA to detect AF or to monitor AF patients even outside the episodes during a normal rhythm. Regarding CHF subgroups in Figures 7d and 7e, we find that both NYHA I–II and NYHA III–IV subgroups significantly differ from the healthy controls in Figure 7f. This demonstrates the potential of DDFA in the early detection of CHF.

## Discussion

This study demonstrates the potential of DDFA for distinguishing between CHF, AF, and healthy subjects using long-term ECG recordings by only utilizing the RRI data, which could be accurately obtained without need for the whole ECG signal. The DDFA features exhibit promising classification accuracy, significantly improving DFA in the multiclass classification task, setting the stage for disease-specific classification.

In general, cardiac diseases have a tendency to lower the (D)DFA scaling exponents. This effect has been found in several studies, including the present one focusing on CHF and AF. Interestingly, decreased scaling exponents are also associated with increasing intensity during physical exercise.^33^^,^^37^ Physiologically, lowering of the scaling exponent corresponds to a decrease in the correlations between the RRIs: α=0.5 corresponds to white noise with no correlations, whereas even lower values indicate anticorrelations, where small and large RR values alternate (in scale s under consideration). As such, these findings are generally not sufficient to distinguish between different cardiac conditions. However, as suggested in this study, a thorough multidimensional examination—accounting for both the scale-dependence and the time- and/or HR-dependence within DDFA—provides valuable information for relatively accurate discrimination between the cardiac conditions.

Previous studies on CHF showed good classification accuracy for PhysioNet datasets when using ensembles of different HRV measures.^8^^,^^9^ Our study has a few clear advantages compared to the previous findings. First, we included substantial additional control data, as well as AF as an additional condition in the control group. Second, we performed robust nested cross-validation for estimating trustworthy classification accuracies. In the study by Chen et al,^9^ 50% train/test split was used, but with such a limited and heterogeneous dataset the robustness of the methodology can be questioned. Based on our nested cross-validation (with different HRV methods), the selection of train/test split has a significant effect on the outcome of the results as seen in the standard deviations shown in Figures 3 and 6.

On the other hand, Liu et al^8^ reported classification from training set yielding the best results from a random forest classifier. However, a big forest with deep enough trees can always overfit into 100% accuracy. In this study , we have focused on an alternative approach with a single powerful measure that also could be utilized further with a combination of other features.

## Study limitations

First, the limited amount of data, especially the relatively small number of subjects, hinders the classification, especially regarding subgroup analysis. In particular, the use of convolutional neural networks is not feasible with this amount of data, even if the problem is otherwise well suited for convolutional neural network architectures. Furthermore, the specificity in detecting exactly CHF or AF is limited because other possible diseases were not taken into account.

As another limitation, the different datasets contain different populations and backgrounds, which might cause the model to learn features specific to these datasets instead of the studied diseases. A major difference in the datasets is the lack of high HRs in the CHF population. This might be caused by possible medications, of which we lack information.

Of note, the HR of CHF patients does not easily increase, since the medical goal is always to increase beta-blocker medication up to the safe maximum dose. This protocol naturally limits physiological performance, especially in the context of CHF with reduced ejection fraction, as both stroke volume and HR remain low. However, the medication has been demonstrated to improve long-term patient prognosis.

Due to limited available metadata for the AF, our confounding factor analysis was limited to CHF and control cases. The effect of age was not statistically significant, which is supported by the previous studies. The DFA-1 α slightly increases with age, whereas the CHF causes significant drops on the scaling exponent.^38^^,^^39^ Similarly, previous studies show slightly increased DFA-1 $\alpha_{1}$ and slightly decreased DFA-1 $\alpha_{2}$ values for men.^40^ This combined with the unbalanced sex distribution in the CHF databases explains the marginally significant effect of sex in the MLR analysis.

Despite these limitations, our DDFA results demonstrate distinctive behavior of CHF and AF subjects. Moreover, the potential of the method is further demonstrated by the consistency of the results regarding the severity of both AF and CHF (Figure 7). The practical possibilities of the analysis are significant. There already exists a modified version of the DDFA algorithm in a wearable consumer-grade HR monitor^32^ for the assessment of training intensity.^33^ This application showcases the possibilities of implementing further algorithms for cardiac risk assessment, such as a CHF detection method, into smart watches and digital HR monitors. Such applications are important for noninvasive remote monitoring to detect problems from simple heart rhythm before hospital visits. Effective guidance to care is crucial because often the first noticeable cardiac symptoms can lead to sudden cardiac death.

## Conclusion

We have shown that recently extended DFA methods yield very good classification accuracies for the detection of CHF from RRIs of long-term ECG measurements. In particular, DDFA overperforms the conventional DFA and has excellent predictive power for CHF, especially in the multiclass classification task. The DDFA approach has significant potential in early detection of both CHF and AF, the latter also from the properties detected during SR.

Overall, the present study shows that improved HRV metrics beyond standard approaches have significant potential for clinical relevance. Whereas deep learning techniques offer another avenue for exploration, methods such as DDFA have strengths in describing relevant features in the characteristics of RRI sequences that reflect different physiological conditions. By combining conventional methods with innovative approaches, we can better understand the complexities of HR dynamics and develop more effective diagnostic tools.

## References

[1] Heidenreich P.A., Bozkurt B., Aguilar D. (2022). 2022 AHA/ACC/HFSA guideline for the management of heart failure: a report of the American College of Cardiology/American Heart Association joint committee on clinical practice guidelines. Circulation.

[2] Malik A., Brito D., Vaqar S. (2024).

[3] Vigen R., Maddox T.M. (2012). Allen LA> Aging of the United States population: impact on heart failure. Curr Heart Fail Rep.

[4] Roth G.A., Mensah G.A., Johnson C.O. (2020). Global burden of cardiovascular diseases and risk factors, 1990– 2019: update from the GBD 2019 study. J Am Coll Cardiol.

[5] Schaffarczyk M., Rogers B., Reer R., Gronwald T. (2022). Validity of the Polar H10 sensor for heart rate variability analysis during resting state and incremental exercise in recreational men and women. Sensors (Basel).

[6] Gilgen-Ammann R., Schweizer T., Wyss T. (2019). RR interval signal quality of a heart rate monitor and an ECG Holter at rest and during exercise. Eur J Appl Physiol.

[7] Seshadri D.R., Bittel B., Browsky D. (2020). Accuracy of Apple watch for detection of atrial fibrillation. Circulation.

[8] Liu Z., Chen T., Wei K., Liu G., Liu B. (2021). Similarity changes analysis for heart rate fluctuation regularity as a new screening method for congestive heart failure. Entropy.

[9] Chen W., Zheng L., Li K., Wang Q., Liu G., Jiang Q. (2016). A novel and effective method for congestive heart failure detection and quantification using dynamic heart rate variability measurement. PloS One.

[10] Peng C.K., Buldyrev S.V., Havlin S., Simons M., Stanley H.E., Goldberger A.L. (1994). Mosaic organization of DNA nucleotides. Phys Rev E Stat Phys Plasmas Fluids Relat Interdiscip Topics.

[11] Peng C.K., Havlin S., Stanley H.E., Goldberger A.L. (1995). Quantification of scaling exponents and crossover phenomena in nonstationary heartbeat time series. Chaos.

[12] Mizobuchi A., Osawa K., Tanaka M., Yumoto A., Saito H., Fuke S. (2021). Detrended fluctuation analysis can detect the impairment of heart rate regulation in patients with heart failure with preserved ejection fraction. J Cardiol.

[13] Molkkari M., Räsänen E. (2018). 2018 Computing in Cardiology Conference (CinC).

[14] Molkkari M., Angelotti G., Emig T., Räsänen E. (2020). Dynamical heart beat correlations during running. Sci Rep.

[15] Pukkila T., Molkkari M., Kim J., Räsänen E. (2022). Reduced RR interval correlations of long QT syndrome patients. Comput Cardiol.

[16] Hernesniemi J.A., Pukkila T., Molkkari M. (2024). Prediction of sudden cardiac death with ultra-short-term heart rate fluctuations. JACC Clin Electrophysiol.

[17] Goldberger A.L., Amaral L.A.N., Glass L. (2000). PhysioBank, PhysioToolkit, and PhysioNet: components of a new research resource for complex physiologic signals. Circulation.

[18] Couderc J.-P. (2010). A unique digital electrocardiographic repository for the development of quantitative electrocardiography and cardiac safety: the Telemetric and Holter ECG Warehouse (THEW). J Electrocardiol.

[19] Couderc J.-P. (2012). The Telemetric and Holter ECG Warehouse (THEW): the first three years of development and research. J Electrocardiol.

[20] Moody G. (August 3, 1999). MIT-BIH Normal Sinus Rhythm Database.

[21] Stein P. (March 3, 2003). Normal Sinus Rhythm RR Interval Database.

[22] Moody G., Mark R. (November 4, 2000). MIT-BIH Atrial Fibrillation Database.

[23] Moody G., Mark R. (1983). A new method for detecting atrial fibrillation using R-R intervals. Comput Cardiol.

[24] Moody G., Swiryn S. (October 20, 2008). Long Term AF Database.

[25] Petrutiu S., Sahakian A.V., Swiryn S. (2007). Abrupt changes in fibrillatory wave characteristics at the termination of paroxysmal atrial fibrillation in humans. Europace.

[26] (October 14, 2008). BIDMC Congestive Heart Failure Database.

[27] Baim D.S., Colucci W.S., Monrad E.S. (1986). Survival of patients with severe congestive heart failure treated with oral milrinone. J Am Coll Cardiol.

[28] Congestive Heart Failure RR (March 3, 2008). Interval Database.

[29] THEW (2008). Healthy Individuals. E-HOL-03-0202-003. http://thew-project.org/Database/E-HOL-03-0202-003.html.

[30] (2008). Definite: Defibrillator in Non-Ischemic Cardiomyopathy Treatment Evaluation TrialE-HOL-03-0401-017. http://thew-project.org/Database/E-HOL-03-0407-017.html.

[31] Räsänen E., Pukkila T., Kanniainen M. (2023). Accurate QT correction method from transfer entropy. Cardiovasc Digit Health J.

[32] Molkkari M, Räsänen E. Inter-beat interval sequence of heart for estimating condition of subject. Patent Pending.

[33] Kanniainen M., Pukkila T., Kuisma J., Molkkari M., Lajunen K., Räsänen E. (2023). Estimation of physiological exercise thresholds based on dynamical correlation properties of heart rate variability. Front Physiol.

[34] Kim J. (2023). Computational Analysis of Complex Beat-to-Beat Dynamics in Heart Cells. Tampere University. https://trepo.tuni.fi/handle/10024/144984.

[35] Chen T., Guestrin C. (2016). Proceedings of the 22nd ACM SIGKDD International Conference on Knowledge Discovery and Data Mining, KDD ’16.

[36] Sun X., Xu W. (2014). Fast implementation of DeLong’s algorithm for comparing the areas under correlated receiver operating characteristic curves. IEEE Signal Process Lett.

[37] Rogers B., Gronwald T. (2022). Fractal correlation properties of heart rate variability as a biomarker for intensity distribution and training prescription in endurance exercise: an update. Front Physiol.

[38] Goldberger A.L., Amaral L.A.N., Hausdorff J.M., Ivanov P.C., Peng C.-K., Stanley H.E. (2022). Fractal dynamics in physiology: alterations with disease and aging. Proc Natl Acad Sci U S A.

[39] Pikkujämsä S. (1999).

[40] Voss A., Schroeder R., Heitmann A., Peters A., Perz S. (2015). Short-term heart rate variability—influence of gender and age in healthy subjects. PLoS One.

