# Supplementary material for: Detection of congestive heart failure from RR intervals during long-term electrocardiographic recordings
Source: Heart Rhythm O2. 2025 Jan 31;6(4):509–18. doi: 10.1016/j.hroo.2025.01.014 (PMC12047600; doi:10.1016/j.hroo.2025.01.014)
Supplement: Supplementary Tables 1-4 [file mmc1.pdf]

Supplementary Material to "Detection of congestive heart failure from RR intervals during long-term ECG recordings"

December 17, 2024

Table 1: Parameter Grid for GridSearch CV, default values of the Scikit-learn and XGboost python packages were used for rest of the parameters. Each of the common resize parameters takes size as tuple containing width and height of the image.

Parameter	Values
**Common Parameters**	
resize__density_size & resize__rest_alpha_size & resize__ex_alpha_size	(2, 2), (2, 4), (2, 8), (2, 16), (4, 2), (4, 4), (4, 8), (4, 16), (8, 2), (8, 4), (8, 8), (8, 16), (16, 2), (16, 4), (16, 8), (16, 16)
PCA n_components	2, 3, 4, 5, 6, 8, 10, 15, 20
XGBoost n_estimators	50, 100, 200, 300
XGBoost gamma	0, 0.1, 0.2
XGBoost max_depth	2, 3, 4, 6, 8
**Binary Classification (2 Classes)**	
XGboost objective	binary:logistic
**Multiclass Classification (3 Classes)**	
XGboost objective	multi:softmax

Table 2: Results of the logistic regression model examining the relationship between the predictor variables and the dependent binary outcome. The table displays the estimated coefficients (Coef.) along with their 95% confidence intervals (CI) in square brackets, the standard error of the estimates (Std. Error), the z-statistics (z) for testing whether the coefficient is significantly different from zero, and the associated p-values (p). Significant predictors are highlighted by p values less than 0.05. Positive coefficients indicate an increase in the log-odds of the outcome with an increase in the predictor, while negative coefficients indicate a decrease.

**Variable**	**Coef. [95% CI]**	**Std. Error**	z	p
Constant	3.9419 [0.944, 6.940]	1.530	2.577	0.010
DFA-1 α_1	-7.7173 [-10.272, -5.163]	1.303	-5.921	< 0.001
Age	0.0308 [-0.003, 0.065]	0.017	1.765	0.078
Sex	-1.5060 [-2.796, -0.216]	0.658	-2.288	0.022

Table 3: Results of the logistic regression model examining the relationship between the predictor variables and the dependent binary outcome. The table displays the estimated coefficients (Coef.) along with their 95% confidence intervals (CI) in square brackets, the standard error of the estimates (Std. Error), the z-statistics (z) for testing whether the coefficient is significantly different from zero, and the associated p-values (p). Significant predictors are highlighted by p values less than 0.05. Positive coefficients indicate an increase in the log-odds of the outcome with an increase in the predictor, while negative coefficients indicate a decrease.

**Variable**	**Coef. [95% CI]**	**Std. Error**	z	p
Constant	-2.5760 [-6.259, 1.107]	1.879	-1.371	0.170
DFA-1 α_2	-1.9691 [-5.431, 1.493]	1.766	-1.115	0.265
Age	0.0533 [0.024, 0.082]	0.015	3.628	< 0.001
Sex	-1.2498 [-2.236, -0.264]	0.503	-2.484	0.013

Table 4: Results of the logistic regression model examining the relationship between the predictor variables and the dependent binary outcome. The table displays the estimated coefficients (Coef.) along with their 95% confidence intervals (CI) in square brackets, the standard error of the estimates (Std. Error), the z-statistics (z) for testing whether the coefficient is significantly different from zero, and the associated p-values (p). Significant predictors are highlighted by p values less than 0.05. Positive coefficients indicate an increase in the log-odds of the outcome with an increase in the predictor, while negative coefficients indicate a decrease.

**Variable**	**Coef. [95% CI]**	**Std. Error**	z	p
Constant	2.7235 [-0.585, 6.032]	1.688	1.613	0.107
DFA-2 α_2	-7.6773 [-10.799, -4.556]	1.593	-4.820	< 0.001
Age	0.0576 [0.027, 0.088]	0.015	3.733	< 0.001
Sex	-1.1582 [-2.285, -0.031]	0.575	-2.015	0.044
